# Clinical Profile and Outcome of Neonatal Pneumothorax: Seven Years of Experience in a Tertiary Care Center

**DOI:** 10.7759/cureus.37625

**Published:** 2023-04-15

**Authors:** Rafat Mosalli

**Affiliations:** 1 Department of Pediatrics, Umm Al-Qura University, Makkah, SAU; 2 Department of Pediatrics, International Medical Center, Jeddah, SAU

**Keywords:** incidence and prognosis, neonatal intensive care unit (nicu), neonatal mortality, survival rate, risk factors, outcomes, saudi arabia, morbidity, pneumothorax, neonate

## Abstract

Background: Neonatal pneumothorax (NP) in neonates is a medical emergency with a significant incidence of morbidity and mortality. There is a paucity of national and regional data about the epidemiological and clinical profiles of pneumothorax.

Aim: The study aim is to identify the demographics, predisposing factors, clinical profiles, and outcomes of NP in a tertiary neonatal care center in Saudi Arabia.

Methods: A retrospective study of all newborns admitted at the neonatal intensive care unit at International Medical Centre, Jeddah, Saudi Arabia, over seven years period between January 2014 and December 2020 was reviewed. A total of 3,629 newborns admitted to the neonatal intensive care unit were included in the study. Data collected included baseline characteristics, predisposing factors, associated morbidities, management, and outcomes of NP. Data were analyzed using the Statistical Package for Social Sciences (SPSS) version 26 (IBM Corp., Armonk, NY).

Results: Of a total of 3,692 included neonates, pneumothorax was detected in 32 neonates with an incidence of 1.02% (ranging from 0.69% to 2%), and 53.1% were males. The mean gestational age was 32 weeks. Our study found that most infants with pneumothorax were extremely low birth weight (ELBW) in 19 babies (59%). The most common predisposing factors were respiratory distress syndrome in 31 babies (96.9%) followed by the need for bag-mask ventilation in 26 babies (81.3%). Twelve newborns (37.5%) with pneumothorax died. Following an analysis of all risk variables, the one-minute Apgar score <5, associated intraventricular hemorrhage, and respiratory support need were shown to be significantly linked with death.

Conclusion: Pneumothorax is not an uncommon neonatal emergency event, especially for ELBW infants, infants requiring respiratory support, or infants with underlying lung disease. Our study describes the clinical profile and affirms the significant burden of NP.

## Introduction

Neonatal pneumothorax (NP) is usually a serious complication of an underlying disease with a high incidence of morbidities and mortality [1]. It happens due to an abnormal accumulation of air between the two pleural layers which subsequently increases the intrathoracic pressure, causing serious negative impacts on the respiratory and cardiovascular systems [1-3]. Early recognition and management of pneumothorax are, therefore, crucial to avoid serious and possibly life-threatening complications.

Clinical examination, transillumination, and chest radiographs, together with the recent utilization of lung ultrasound are used to make an effective diagnosis and in the timely management of pneumothorax [4,5]. National data from Saudi Arabia and developing countries were quite limited. Consequently, we are conducting a retrospective study to describe the incidence, predisposing factors, complications, and outcomes of NP. Our rationale is to provide local data on this condition that might help in building an informative national registry toward establishing standardized targeted care for the neonatal population at risk.

## Materials and methods

Study setting

The Neonatology Section with 25 ventilated Neonatal Intensive care (NICU) beds, International Medical Center (IMC) is considered one of the largest and busiest private sectors in the Western region and kingdom that provides a tertiary care level for the neonatal population. In addition, the level 1 and regular nursery can accommodate up to 35 beds. All levels are usually operated with an average of 90% monthly occupancy rate. Total deliveries from 2014 to 2020 were 27,417 inborn, out of which 3,629 (13%) were admitted to the NICU.

Study subjects

All neonates who were admitted to the neonatal unit and were diagnosed with Pneumothorax were included within seven years from January 1, 2014, and December 31, 2020. Neonates with major congenital anomalies and metabolic disorders were excluded. Otherwise, all admitted neonates to the NICU at the IMC from 2014 to 2020 were included in the study.

Study design

A retrospective cohort study based on seven-year data of neonates with pneumothorax. These infants were identified from the neonatal electronic medical record and delivery logbook of the neonatal intensive care and maternity unit. Patients’ charts were retrieved and data on maternal and neonatal demographics, incidence, clinical profile, management, and outcome were extracted. The following neonatal and maternal variables were extracted: Maternal age, prenatal corticosteroid use, mode of delivery, gender, gestational age (GA), birth weight, chorioamnionitis, Apgar scores at one and five minutes of age, length of stay in the NICU (days), respiratory distress syndrome (RDS), use of surfactant, bronchopulmonary dysplasia (BPD), hemodynamically significant patent ductus arteriosus (HSDA), intraventricular hemorrhage (IVH), retinopathy of prematurity (ROP), necrotizing enterocolitis (NEC), early and late-onset sepsis and survival at discharge. After IMC Research Ethics Board approval was granted, the medical records of eligible cases were extracted and electronically pooled and reviewed by trained persons.

Assumed predisposing factors

Maternal and neonatal characteristics that could lead to pneumothorax were identified in priori and extracted from the database. These data included: GA, birth weight, sex, Apgar score <5 at five minutes, cesarean section (CS), prolonged rupture of membrane ( PROM) >24h, antenatal steroid administration, the need for bag-mask ventilation (BMV), use of continuous positive airway pressure (CPAP) or noninvasive positive pressure ventilation (NIPPV), mechanical ventilation (MV), use of surfactant, RDS and other underlying lung disease such as atelectasis and meconium aspiration, early and late-onset sepsis.

Outcomes and associated illnesses include the length of stay, survival, and accompanying disorders such as BPD, NEC, IVH, ROP, and pulmonary hemorrhage. Description of pneumothorax includes the timing of occurrence, severity, laterality, treatment intervention, and time to resolve.

Data management and analysis

Data entry and processing were carried out on the IBM-Statistical Package for Social Sciences (SPSS) version 26 (IBM Corp., Armonk, NY). We performed descriptive analysis for risk and associated illness. Additional analysis to examine the association between the survival of pneumothorax and predisposing factors, underlying conditions, length of stay, and management course was conducted using the Chi-square test and the Mann-Whitney U test for inferential analysis. A P-value of <0.05 indicated statistical significance.

## Results

This study aimed to investigate the incidence, predisposing factors, survival, and outcomes associated with NP.

Incidence of pneumothorax

Of a total live birth of 27,417 newborns during the study period, 3,629 neonates were admitted to level three NICU of whom 32 neonates (31 preterms and only one term) were diagnosed with NP and met the inclusion criteria, giving an overall incidence of 1.02%.

Predisposing factors

Our study included 32 subjects, and the mean maternal age was 29 ± 5 (19-39) with 22 mothers (68.8%) having CS delivery and 10 (31.3%) having normal spontaneous vaginal delivery (NSVD). The mean GA was 32 ± 9 weeks (23-41). Seventy-five percent of the mothers had dexamethasone injections during the third trimester. The included newborns were 15 females and 17 males with a percentage (46.9%, and 53.1%, respectively). Most of the neonates were extremely low birth weight (<1,000 g), 19 (40.6%) newborns, eight (25%) newborns were between 1,000 and 1,499 g, five (15.6%) newborns were between 1,500 and 2,499 g., and only six (18.8%) newborns had normal birth weight between 2,500 and 4,000 g. The average one-minute Apgar score was 6 ± 2 (0-9) and the five-minute APGAR score was 8 ± 1 (3-10) (Table 1).

**Table 1:** Baseline characters of the neonates diagnosed with pneumothorax (n=32). PROM: Prolong rupture of membrane; CS: Cesarean section; NSVD: Normal spontaneous vaginal delivery.

Parameter		Number (%)
Maternal age, years		29 ± 5 (19-39)
PROM		4 (12.5%)
Steroid use		24 (75%)
MODE	CS	22 (68.8%)
	NSVD	10 (31.3%)
Gender	Female	15 (46.9%)
	Male	17 (53.1%)
Birth Weight (gram)	<1000	13 (40.6%)
	1000- 1499	8 (25%)
	1500- 2499	5 (15.6%)
	2500- 4000	6 (18.8%)
1 Minute APGAR Score	7-10	17 (53%)
	4-6	10 (32%)
	0-3	5 (15%)

**Figure 1:**
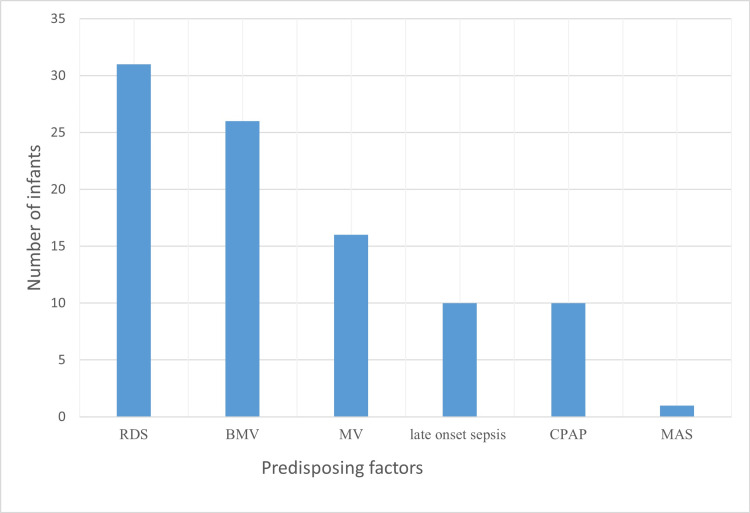
Number of infants with predisposing factors of pneumothorax (n = 32). RDS: Respiratory distress syndrome, BMV: Bag-mask ventilation; MV: mechanical ventilation; CPAP: Continuous positive airway pressure; MAS: Meconium aspiration syndrome.

The most common predisposing factors of pneumothorax in our sample was RDS in 31 (96.9%) neonates, followed by BMV during resuscitation in 26 (81.3%) neonates, 16 (50%) neonates who require mechanical ventilation, 10 (31%) neonates with CPAP and 10 (31%) neonates with late onset sepsis, and only five (15.6%) neonates had atelectasis (Figure 1).

Accompanying disorders and outcomes

**Figure 2:**
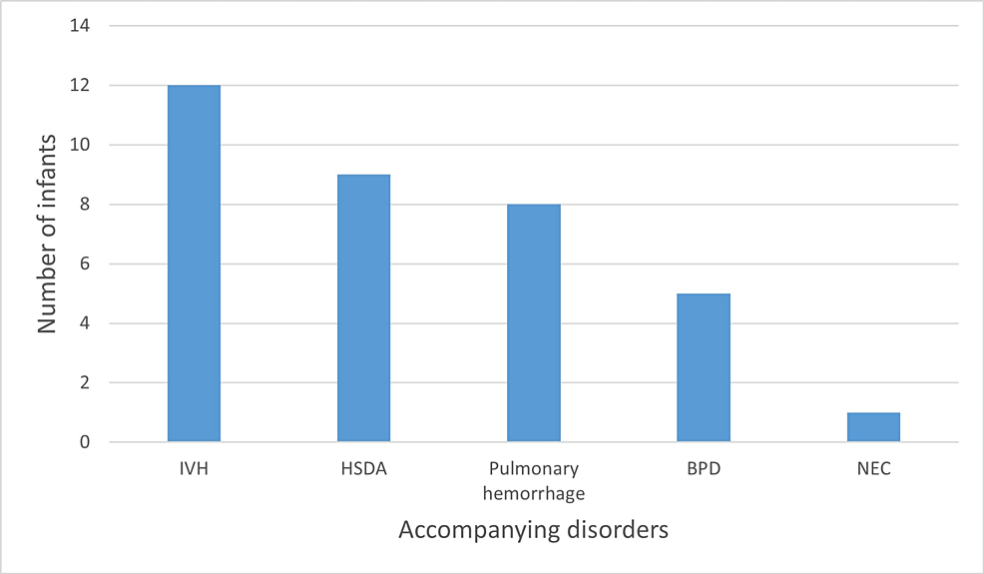
Accompanying disorders in neonates with pneumothorax (n=32). IVH: intraventricular hemorrhage, HSDA: hemodynamically significant patent ductus arteriosus; BPD: bronchopulmonary dysplasia; NEC: necrotizing enterocolitis.

The most common accompanying disorders were IVH in 12 (37%) neonates followed by HSDA in nine (28%) newborns and pulmonary hemorrhage in eight (25%) neonates (Figure 2). The description of pneumothorax, management with an overall survival rate of 62.5% is presented in Table 2.

**Table 2:** Description of pneumothorax and management outcomes.

Parameter		Frequency (%)
Onset , hours	<72h	16 (50%)
	>72h	16 (50%)
Time resolved , hours		14 ± 16 (2-67)
Treatment of Air leak	Chest Tube	20 (62.5%)
	Needling	7 (21.9%)
	Expectant	5 (15.6%)
Site	Bilateral	6 (18.8%)
	Unilateral	26 (81.3%)
Survival	No	12 (37.5%)
	Yes	20 (62.5%)

Table 3 shows the relation between the different parameters and survival status. There was a significant correlation between survival outcome and one-minute Apgar, respiratory support, and IVH with P-value = 0.025, 0.046, and 0.029, respectively. Although the number of cases survived was higher in CS delivery than in NSVD, we however found a non-significant relation between survival status and each of the following: mode of delivery, gender of the baby and birth weight with P-value = 0.844, 0.55, and 0.673, respectively, the rest of the maternal and neonatal parameters had non-significant correlations with survival outcome with non-significant P-value (Table 3).

**Table 3:** Survival in association with predisposing factors, and associated conditions (n=32). *Chi-square test was used. **Mann-Whitney U test was used. CS: cesarean section; NSVD: normal spontaneous vaginal delivery, MV: mechanical ventilation; HFNC: high flow nasal cannula; HFOV: high flow oscillatory ventilator; NCPAP: nasal continuous positive airway pressure NIPPV: noninvasive positive pressure ventilation.

Parameter		Outcome		P-value*
		Died	Survived	
MODE	CS	8 (36.4%)	14 (63.6%)	0.844
	NSVD	4 (40%)	6 (60%)	
Gender	Female	3 (20%)	12 (80%)	0.055
	Male	9 (52.9%)	8 (47.1%)	
Birth Weight, gram	<1000	6 (46.2%)	7 (53.8%)	0.673
	1000-1499	3 (37.5%)	5 (62.5%)	
	1500-2499	2 (40%)	3 (60%)	
	2500-4000	1 (16.7%)	5 (83.3%)	
Gestational age , weeks		28 ± 4	34 ± 11	0.071**
1 Minute APGAR Score		5 ± 2	7 ± 2	0.025**
5 Minute APGAR Score		7 ± 2	8 ± 1	0.091**
Resuscitation	No	1 (16.7%)	5 (83.3%)	0.242
	Yes	11 (42.3%)	15 (57.7%)	
Respiratory support		0 (0%)	1 (100%)	0.046
	MV	6 (46.2%)	7 (53.8%)	
	HFNC	2 (100%)	0 (0%)	
	HFOV	3 (75%)	1 (25%)	
	NCPAP	1 (12.5%)	7 (87.5%)	
	NIPPV	0 (0%)	4 (100%)	
Number of Surfactant administration	0	1 (20%)	4 (80%)	0.776
	1	3 (33.3%)	6 (66.7%)	
	2	5 (45.5%)	6 (54.5%)	
	3	3 (42.9%)	4 (57.1%)	
Respiratory distress syndrome	No	0 (0%)	1 (100%)	0.431
	Yes	12 (38.7%)	19 (61.3%)	
Atelectasis	No	11 (40.7%)	16 (59.3%)	0.379
	Yes	1 (20%)	4 (80%)	
Pneumonia	No	12 (38.7%)	19 (61.3%)	0.431
	Yes	0 (0%)	1 (100%)	
Sepsis	No	11 (35.5%)	20 (64.5%)	0.19
	Yes	1 (100%)	0 (0%)	
Pulmonary haemorrhage	No	8 (32%)	17 (68%)	0.225
	Yes	4 (57.1%)	3 (42.9%)	
Necrotizing enterocolitis	No	12 (38.7%)	19 (61.3%)	0.431
	Stage1	0 (0%)	1 (100%)	
Hemodynamically significant patent ductus arteriosus	No	7 (29.2%)	17 (70.8%)	0.092
	Yes	5 (62.5%)	3 (37.5%)	
Intraventricular haemorrhage	Grade I-II	0 (0%)	4 (100%)	0.029
	Grade III-IV	5 (83.3%)	1 (16.7%)	
	Complicated	0 (0%)	2 (100%)	
	No	7 (35%)	13 (65%)	
Early onset sepsis	No	11 (35.5%)	20 (64.5%)	0.19
	Yes	1 (100%)	0 (0%)	
Late onset sepsis	Complicated	1 (50%)	1 (50%)	0.526
	No	9 (42.9%)	12 (57.1%)	
	Yes	2 (22.2%)	7 (77.8%)	
Length of stay , days		13 ± 22	6 ± 6	0.826**
Time resolved , days		22 ± 28	11 ± 7	0.784**
Treatment of Air leak	Chest Tube	10 (50%)	10 (50%)	0.206
	Needling	2 (28.6%)	5 (71.4%)	
	No	0 (0%)	4 (100%)	
	Spontaneous	0 (0%)	1 (100%)	
Site	Bilateral	3 (50%)	3 (50%)	0.483
	Unilateral	9 (34.6%)	17 (65.4%)	

## Discussion

Pneumothorax is more common in newborns than in any other age group and is linked to higher death and morbidity rates, especially in premature infants [3,6]. The incidence rate of NP ranges between 0.5% and 3% in the total population of live births [1-3,7-10]. The incidence is higher in hospitalized sick newborns admitted to NICU and varies from 1.5% to 20% [2,3,6-8,11-15]. In our study, the incidence of NP from total live births was 0.14% while the incidence for newborns admitted to NICU was 1.02% which is also lower than a comparable study conducted in Saudi Arabia at three years period with a reported incidence of 3.9% [16]. The variation in the reported incidence of NP could be attributed to the neonatal population type, the institution’s variations in epidemiological and clinical profile, and the management approach, especially at the time of initial resuscitation from the time of delivery.

Male sex, low birth weight neonates, preterm, neonates delivered through cesarean section, the presence of underlying lung diseases such as RDS, and meconium aspiration necessitating postpartum resuscitation are all known predisposing factors for NP [1,10,13,17,18]. The demographic profiles of our study were consistent with previous studies in which most infants with pneumothorax were male, premature babies <32 weeks, and neonates with very low birth weight (VLBW)<1,500 g, low one minute Apgar score and need for positive pressure ventilation, presence of concurrent neonatal RDS.

NP in our study mostly occurred in the first 72 h of life (50%) which also corresponded with previous literature [1,8,10,19-21]. Like in most previous studies, the position of the NP in our study was mostly unilateral (81.3%) [2,6] and only six newborns (18.8%) had bilateral NP.

NP management strategies are not completely understood. For asymptomatic patients who have minor PN without underlying pulmonary disease, no surgical intervention is needed, and expectant management should be employed [22,23]. Needle aspiration, chest tube insertion, and ventilator support should be considered if the patient develops signs of respiratory distress and hemodynamic instability [24]. In our study, treatment of air leaks was needed in most of the cases, using a chest tube in 20 cases (62.5%), needling in seven cases (21.9%), and expectant management (15.6%). The mean time resolved in our result was 14 ± 16 days (2-67) (Table 2). 

Many studies have shown that NP rates rise with neonatal RDS, BMV, and ventilation-associated lung injuries in terms of barotrauma and/or volutrauma was documented as the major cause of NP [1,2,16,25,26]. This is consistent with our study in which the RDS was the most prevalent predisposing factor in 97% followed by BMVs 81%, mechanical ventilation in 50% and use of CPAP in 31% (Figure 1). These data imply that there is continuous demand by neonatologists to practice protective lung strategies to reduce related complications and improve patient outcomes.

The most associated morbidities with NP were IVH, BPD, HSDA, and pulmonary hemorrhage [6,16]. This is to some extent consistent with our study in which IVH was the most associated morbidity in 37.5% of neonates, followed by HSDA (28%) and Pulmonary hemorrhage (25%) (Figure 2).

Despite all attempts at making an early diagnosis and effective management of NP, the mortality rate is still high [3,16].In our study, the mortality rate was relatively high (37.5%). It is comparable with previous studies as it varied from 20% to 38% [6,16,27-29]. In our study, the mortality rate linked to low Apgar score in the first minute, need for respiratory assistance, and IVH with P-value = 0.025, 0.046, and 0.029, respectively (Table 3). In general, 62.5% of our surviving neonates had full recovery at discharge and without major neonatal morbidities.

Though our study has some limitations, including data collection, a single-center study with a small sample size, it is, however, the first national retrospective study with an extended period over seven years that includes an extended, indeed wide category of demographic and risk factors spanning all gestation ages. In addition, this study was conducted in the private sector which could serve as baseline comparative data for the public sector population. Overall, this data could help the upcoming nationwide studies to better design a multicenter prospective trial to further explore the burden of NP at different institutional sectors for better delivery of health care resources.

## Conclusions

NP is an undesirable neonatal emergency condition with a complicated clinical course that could be associated with significant major morbidities such as IVH and HSDA, especially in extremely premature infants. Early identification of high-risk newborns together with standardized and timely fashion management guidelines is quite crucial and could improve survival and outcome.
